# Upscaling irradiation protocols of *Aedes albopictus* pupae within an SIT program in Reunion Island

**DOI:** 10.1038/s41598-024-62642-7

**Published:** 2024-05-27

**Authors:** Lucie Marquereau, Hanano Yamada, David Damiens, Antonin Leclercq, Brice Derepas, Cécile Brengues, Brice William Dain, Quentin Lejarre, Mickael Proudhon, Jeremy Bouyer, Louis Clément Gouagna

**Affiliations:** 1 UMR Mivegec (Maladies Infectieuses et Vecteurs: Écologie, Génétique, Évolution et Contrôle), IRD-CNRS-Univ. Montpellier, Représentation IRD la Réunion – PTU, 97495 Sainte Clotilde Cedex, La Réunion, France; 2 Insect Pest Control Laboratory, Joint FAO/IAEA Programme of Nuclear Techniques in Food and Agriculture, IAEA Vienna, Wagramer Strasse 5, 1400 Vienna, Austria; 3 ASTRE, CIRAD, INRAE, University of Montpellier, 34398 Montpellier, France; 4 ASTRE, CIRAD, INRAE, University of Montpellier, Plateforme Technologique CYROI, Sainte-Clotilde, La Réunion, France; 5 UMR Mivegec, IRD-Délégation Régionale Occitanie, 34394 Montpellier, France

**Keywords:** Sterile insect technique, Upscale, Irradiation method, *Aedes albopictus*, Biotechnology, Ecology

## Abstract

The implementation of the sterile insect technique against *Aedes albopictus* relies on many parameters, in particular on the success of the sterilization of males to be released into the target area in overflooding numbers to mate with wild females. Achieving consistent sterility levels requires efficient and standardized irradiation protocols. Here, we assessed the effects of exposure environment, density of pupae, irradiation dose, quantity of water and location in the canister on the induced sterility of male pupae. We found that the irradiation of 2000 pupae in 130 ml of water and with a dose of 40 Gy was the best combination of factors to reliably sterilize male pupae with the specific irradiator used in our control program, allowing the sterilization of 14000 pupae per exposure cycle. The location in the canister had no effect on induced sterility. The results reported here allowed the standardization and optimization of irradiation protocols for a Sterile Insect Technique program to control *Ae. albopictus* on Reunion Island, which required the production of more than 300,000 sterile males per week.

## Introduction

The Sterile Insect Technique (SIT) is a biological and species-specific method of insect pest control. It relies on releasing continuous and overflooding numbers of sexually competitive sterile insects, usually males, into a wild population of the same species. When wild females mate with sterile males, their reproduction is blocked, thus suppressing or even eradicating the target population over successive generations^1^. Over the last decades, successful SIT programs have suppressed populations of various pests of veterinary and agricultural importance, such as fruit flies, tsetse flies, screw worms and moths^2^. However, the success of the SIT against mosquitoes, a medically important pest, has varied depending on factors such as species, sterilization methods, and numerous other circumstances^3,4^. In recent years, there has been renewed interest in the SIT for mosquitoes, particularly for *Aedes* species, due to increased arbovirus outbreaks and the rapid spread of Aedes mosquitoes into new territories^5–7^. While the SIT programs and technical components are still under development for mosquitoes^8–11^, and are still relatively small compared to programs for plant pests, significant progress has been made more recently as reviewed in Vreysen et al.^12^. This progress is crucial for the integrated management of Aedes-borne viral diseases^13,14^.

The implementation potential of the SIT for mosquito management depends mainly on the capacity to produce sterile males. This requires efficient mass-rearing facilities, with cost-effective methods for insect production, as well as a reliable and reproducible sterilization method with minimal negative effects on male mating competitiveness and other biological quality parameters. Several components are needed to support the testing and implementation of the SIT including baseline data collection to provide information on the biological and ecological parameters of the species, methods for mass rearing, sex separation, sterilization, packing, transport, release and quality control^8–10,15–18^. Continuous research is also needed for the optimization and standardisation of each step of the technique from small-scale field trials to the operational phase of the SIT programs.

In the SIT, the sterilization step is critical and has been described in numerous studies since 1960^3^. Technical parameters, methods of the sterilization and biological features of *Aedes* species can significantly affect the sterility of males. Exposure to ionizing radiation (gamma Co-60 or Cs-137, X-ray) is currently the method of choice for rendering insects reproductively sterile^19^. It has been shown that X-ray provides a practical and effective alternative to gamma-ray for sterilization of insects, are often less expensive, and are subjected to less stringent regulations^20–22^. Ionizing radiation induces dominant lethal mutations in the chromosomes of germ cells of the insect^19^, significantly affecting the fertility of males^22–25^. The higher the absorbed dose, the higher the sterility, and the higher the risk of off-target somatic damage occurring, which can negatively impact insect quality^26^. Thus, radiation dose presents a trade-off between effective sterilization and the quality of the sterile males^27^. Mating competitiveness, survival and flight ability of sterile males need to be as close as possible to the wild population^28^. The optimum dose to ensure sufficient sterility without excessive loss in quality depends on each strain, and each facility-specific set-up. For example, in some facilities, the optimum dose for *Aedes albopictus* is reported to be between 35 and 40 Gy whereas it is 50 Gy for *Aedes aegypti*^20,24,29,30^. Other facilities require 55 Gy and 70 Gy for the two species respectively^21,31,32^. Dose–response is also affected significantly by atmospheric conditions during irradiation, and particularly oxygen levels have been shown to have significant impact on irradiation outcome^25,33^. For *Ae. albopictus* and *Ae. aegypti*, studies have shown a significant radioprotective effect of oxygen depletion during irradiation procedures^25^. Biological characteristics of *Aedes spp.* can also impact radiosensitivity, such as insect life stage and pupal age during irradiation. The maturation of insect reproductive organs and also germ cell development changes with life stage and age, affecting sensitivity to radiation induced somatic damage^26^. A strong negative correlation has been shown between pupal age and radiosensitivity for *Ae. aegypti*^25^ and *Ae. albopictus*^34^. For the SIT, it is therefore important to irradiate with a dose sufficient to sterilize the oldest pupae to ensure that no sub-sterile males remain after irradiation^33^. Other factors such as the nutritional state of pupae, effects of pre-conditioning stress factors, and diapause may additionally influence radioresistance in mosquitoes and have yet to be assessed for *Aedes spp*^19–22^.

Studies on the baseline data collection of pupae sterilization have, for the most part, been performed on small samples, i.e. consisting of a few hundred individuals, and sometimes less. In order to upscale the SIT for field trials on La Reunion Island, the production of around 300 000 sterile males per week is needed^6^. Thus, the capacity to irradiate a larger number of pupae in bulk is an essential parameter to assess. To date, no method for large scale bulk-irradiation of pupae has been described. Currently, there are few countries working on mass pupae sterilization (China, Italy, Singapore, USA), but there is no detailed information available on the methodologies used in these programs. Effective and standardized mass irradiation methods are thus needed.

As part of the up-scaling process towards a SIT pilot trial against *Ae. albopictus* on La Reunion^6^, the aim of the present study was to develop an efficient upscaled irradiation protocol for a weekly production of 300 000 sterile males of *Ae. albopictus* with the specificities of the only available irradiator on the island. Using available resources of La Reunion, and according to previous studies to determine optimum factors of sterilization for the species, we assessed the effects of exposure environment, pupae density, quantity of water and irradiation dose on the dose–response of *Ae. albopictus* male pupae. We also evaluated the effect of sample location within the irradiation canister to ensure a consistent and reproducible sterilization method. The long-term objective of this work, which occurred in parallel with the field pilot testing of the SIT on La Reunion, was to acquire sufficient information to develop protocols for the effective and reliable upscaled irradiation of mosquito pupae.

## Results

### Effect of exposure environment and pupae density

**Figure 1:**
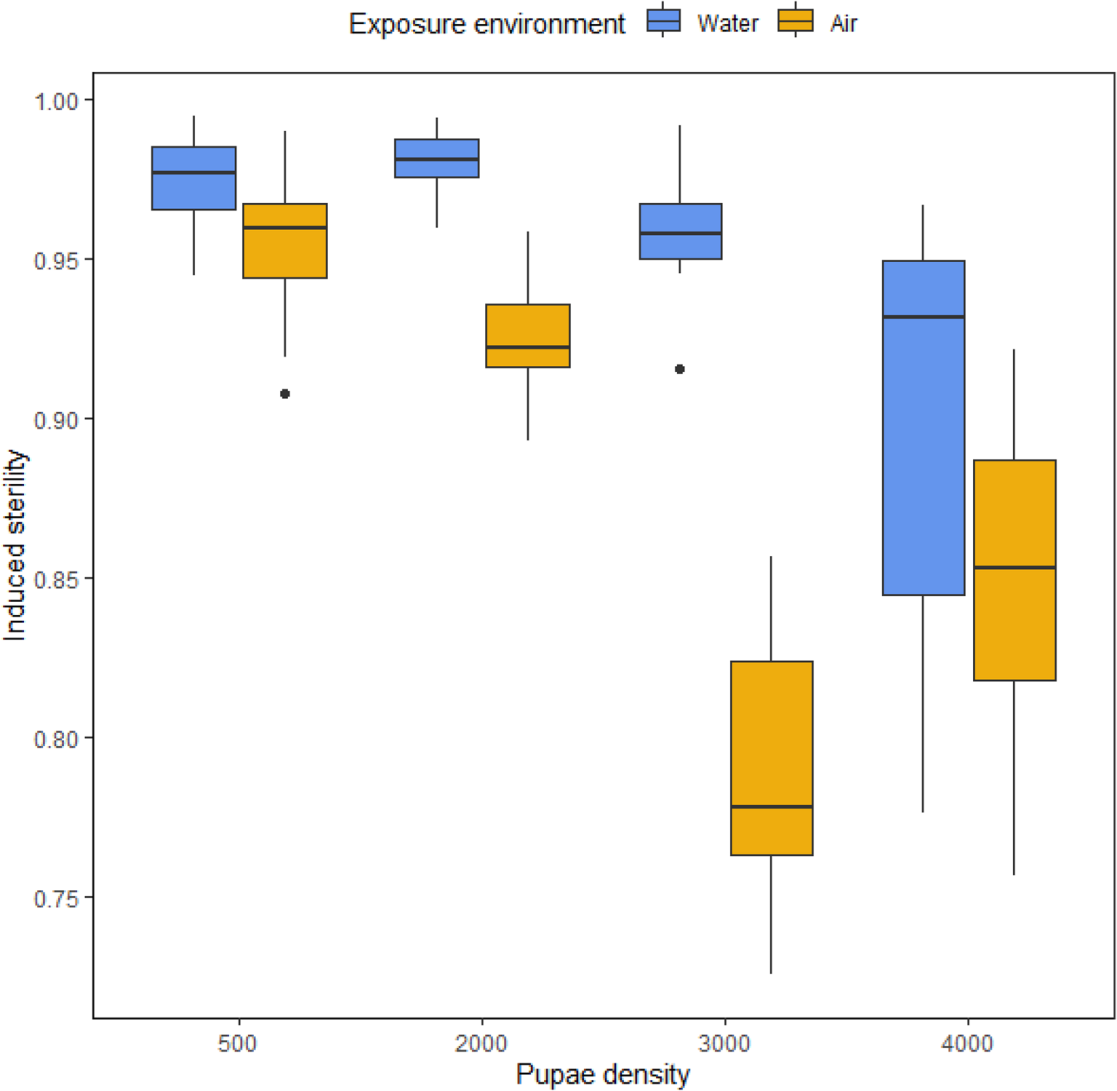
Induced sterility of *Ae. albopictus* irradiated in water or in air increasing pupae densities. The box plot shows the median and upper and lower quartiles.

Mean induced sterility spanned from 0.977 in batches of 500 pupae to 0.931 with 4000 pupae following exposure in water, whereas mean induced sterility spanned from 0.959 in batches of 500 pupae to 0.853 with 4000 pupae following exposure in air (Fig. 1). The best model included all fixed effects and the interaction between exposure environment and pupae density. There was a significant effect of the exposure environment (*P* = 0.0028, Table 1), where irradiation in water showed higher induced sterility than irradiation in air. There was no difference between the induced sterility of 500 pupae and 2000 pupae either in water (*P* = 0.3155) or in air (*P* = 0.1005). For larger pupae densities (3000 and 4000), increased pupae density reduced induced sterility (Table 1) and we also observed more variability in induced sterility, especially for irradiation in air. Finally, a significant interaction between pupae density and exposure environment was only observed at a density of 3000, where water led to an increased induced sterility than air (*P* < 10^–3^).

**Table 1:** Fixed-effects coefficients of a mixed-effect binomial model of the impact of environment and pupae density during irradiation at 35 Gy on the induced sterility of *Aedes albopictus* (93 observations).

Fixed effects	Value	Std. error	t-value	*P*-value
Intercept	0.9912540	0.01448649	68.42612	0.0000
Environment air	− 0.0520944	0.01678341	− 3.10392	0.0028***
Density 2000	0.0122901	0.01714423	0.71686	0.4759
Density 3000	− 0.0290617	0.01355612	− 2.14381	0.0356*
Density 4000	− 0.1020294	0.01316343	− 7.75098	0.0000***
Environment air:density 2000	0.0234476	0.02564927	− 0.91416	0.3639
Environment air:density 3000	0.1148625	0.02625381	− 4.37508	0.0000***
Environment air:density 4000	0.0026039	0.01959871	− 0.13286	0.8947

*P ≤ 0.05 ; ***P ≤ 0.01.

### Effect of dose and water quantity

**Figure 2:**
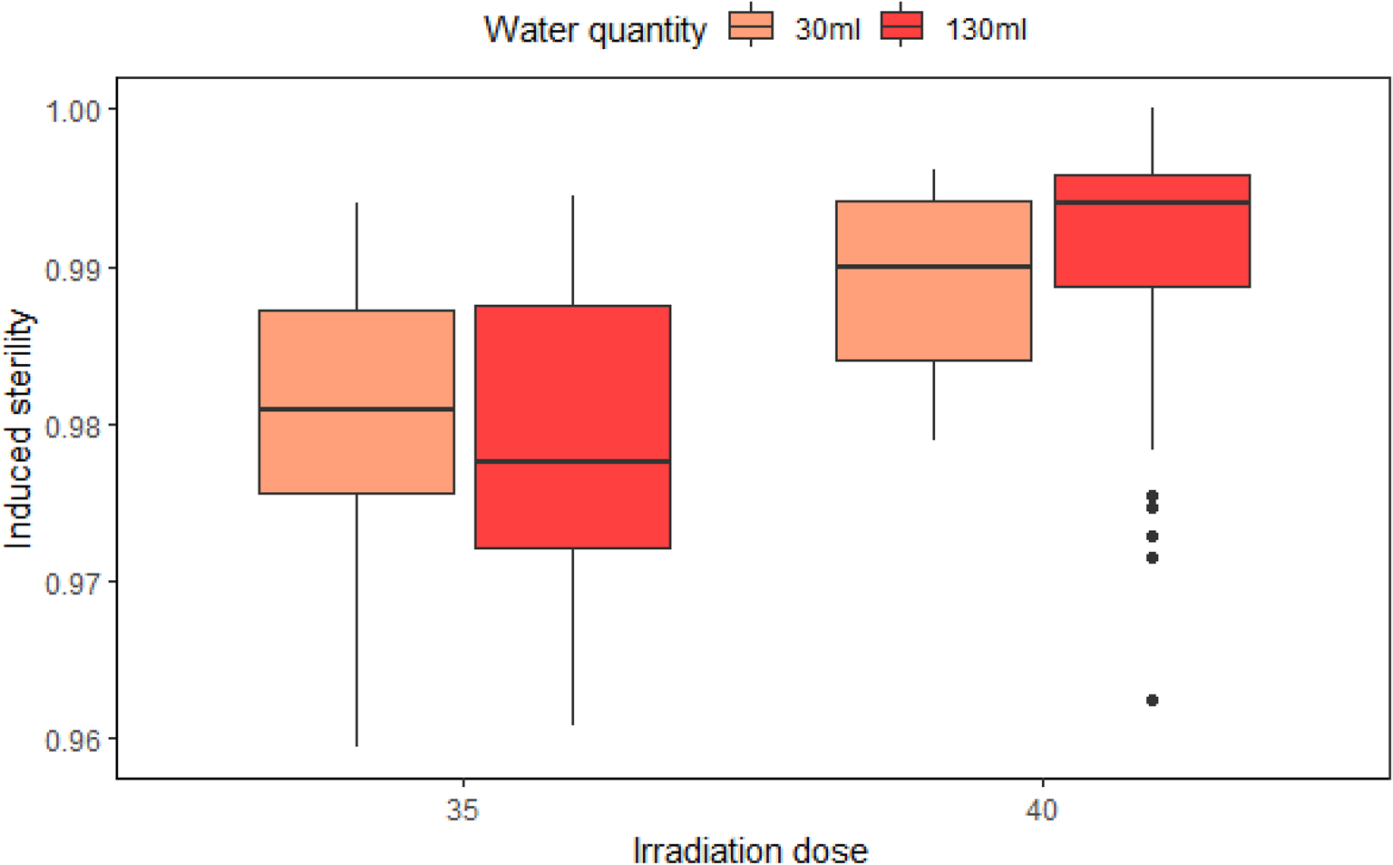
Induced sterility of *Ae. albopictus* following irradiation at 35 Gy or at 40 Gy depending on quantity of water during the irradiation of 2000 pupae in water. The box plot shows the median and upper and lower quartiles.

The best model included all fixed effects without the interaction between irradiation dose and water quantity. The mean induced sterility was higher following irradiation at 40 Gy (*P* = 0.0000, Table 2) and showed less variability, as compared to irradiation at 35 Gy. There was no effect of the quantity of water, however the mean induced sterility (and upper and lower quartile) was higher with more water (Fig. 2).

**Table 2:** Fixed-effects coefficients of a mixed-effect binomial model of the impact of irradiation dose and water quantity on the hatch rate of eggs of *Aedes albopictus* (165 observations).

Fixed effects	Value	Std. Error	t-value	*P*-value
Intercept	0.9795565	0.002341964	418.2629	0.0000***
Dose 40 Gy	0.0108060	0.001871100	5.7752	0.0000***
Water 130 ml	0.0011102	0.001804061	0.6154	0.5394

***P ≤ 0.01.

### Optimization of pupae density

Mean induced sterility of *Ae. albopictus* ranged from 0.994 for pupae density of 500 to 0.987 for pupae density of 2500 (Supplementary Fig. S1). There was no difference in the induced sterility between pupae irradiated at a density of 500 and 2000 (*P* = 0.9399) but increasing the density to 2500 reduced the induced sterility (*P* = 0.0496). When all extreme data belonging to one particular repetition were removed from the analysis, the difference was even more significant (*P* = 0.0021).

### Effect of the pupae location within the irradiation canister

**Figure 3:**
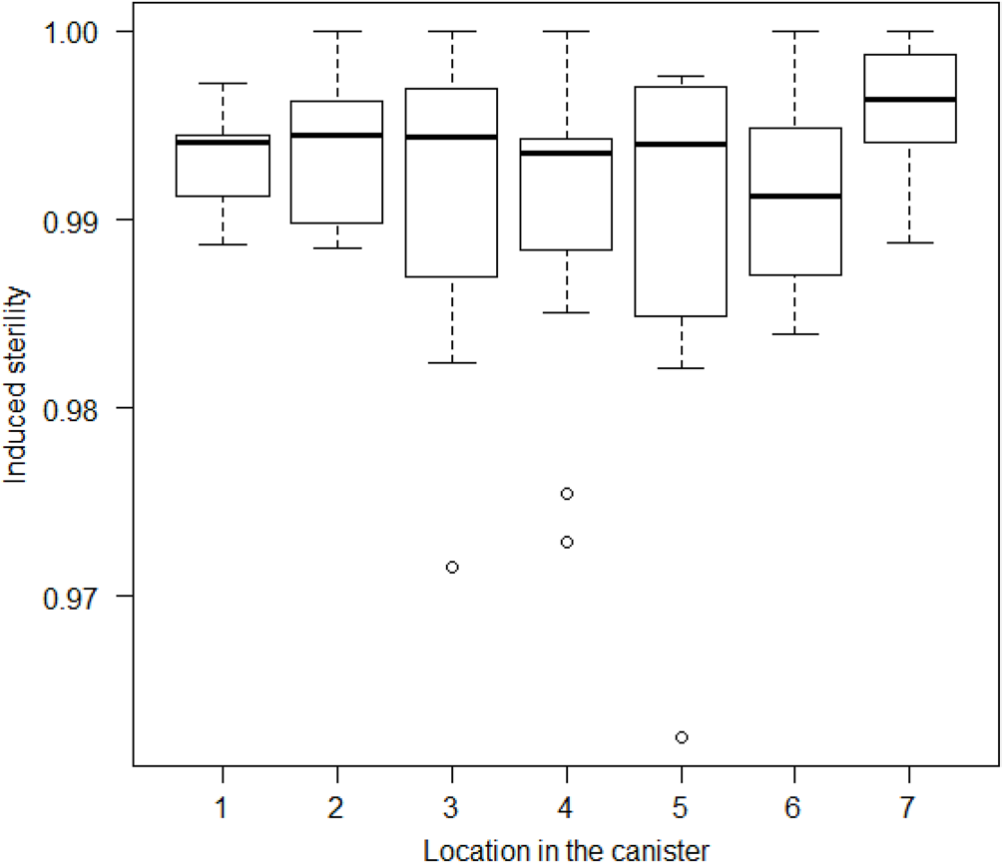
Induced sterility of *Ae. albopictus* following the exposure at various locations in the canister for the irradiation of 2000 pupae in 130ml of water at 40 Gy in a full canister. The box plot shows the median and upper and lower quartiles.

Mean induced sterility ranged between 0.991 and 0.996 in samples placed throughout the canister and showed more variability in the central locations for a fixed dose of 40 Gy in 130 ml of water (Fig. 3). The location of pupae in the canister did not affect the induced sterility of *Ae. albopictus*, except for the seventh location, at the top of the canister (*P* = 0.0083), when location 4 was taken as a reference in the analysis. Central positions in the canister between 3 and 6 showed more variability in induced sterility in adults. There was no difference between locations 1 and 7. However, when removing extreme data from the analysis (all belonging to the same repetition as in experiment 3), no difference was observed for all positions in the canister (*P* = 0.526).

## Discussion

The series of experiments reported here have shown that there are different factors that affect induced sterility in *Ae. albopictus,* which must be taken into consideration when developing a routine irradiation protocol for pupae during an SIT control program.

Exposure in water during pupae irradiation led to higher induced sterility than exposure in air in our experiment. Access to oxygen could explain this result. Generally, mosquito pupae seem to be bimodal breathers, obtaining oxygen via their respiratory trumpets directly from the air when they float on the water surface^35,36^, and via diffusion of dissolved oxygen (DO) from the water through their cuticle when submerged^37,38^. Studies have shown depletion of DO in water by *Ae. albopictus* pupae. When they are enclosed in water with no air bubble at a density of 100 pupae/ml, the pupae depleted the water of DO reaching 50% within 5–6 min and 0.1% DO within 20–25 min^33^. When pupae are surrounded by an oxygen-poor environment, a protective effect against irradiation effects was demonstrated (water with < 0.5% DO), showing a lower induced sterility in pupae^25,33^, and similar effects were observed for the irradiation of adults in nitrogen^39^. In our conditions, the water containing the pupae was renewed just before the treatment and the petri dish offered a large surface area, which allows all pupae to float in a monolayer (except for the density of 4000 pupae) and breathe the ambient air above the water suggesting that pupae had enough oxygen not to incur protection against irradiation. On another hand, when pupae were treated in air, respiration could have been impaired by the fact that pupae were compressed together while lying on the side, especially in treatments with a large number of pupae that could avoid respiration with the trumpets. Moreover, the pupae were not totally dry and were covered in a film of water that could still create small pockets of hypoxia. A low density of pupae per ml may optimize the access to the oxygen for pupae, both in water via cuticular respiration, and in air, via access to the surface, minimizing the protective effect of hypoxia. Further experiments are needed to evaluate differences between conditions in air and conditions in water during pupae irradiation on resulting induced sterility and also the effects of handling pupae in bulk for dry conditions in the context of large-scale irradiation.

The number of pupae that can be irradiated in one irradiation event depends on the irradiator’s specificities, and self-contained units are limited by the chamber, or canister size. Each facility requires the optimization of methods depending on the irradiator available. To our knowledge, no irradiation study had been previously performed on the effects of pupae density during irradiation. We demonstrated a decrease of induced sterility with the increase in pupal density, when pupae density exceeded 3000/petri dish. The larger the sample size and volume of material, the more heterogeneous the absorbed dose. A biological hypothesis could be that with higher densities of pupae, more O_2_ is depleted in the surrounding water, providing some radioprotection in the pupae. These two hypotheses could be investigated respectively be studying effect of different location in the petri dishes and effect of quantity of water (with measuring the dissolved oxygen) on the induced sterility. In our experiment, no effect of the density on the induced sterility was observed for irradiation up to 2000 pupae, which clearly appeared to be the best pupae density for pupae irradiation at 35 Gy.

In our experiment, we showed that irradiation of pupae in a larger quantity of water provided no significant effect on induced sterility in the adults, at both 35 Gy or at 40 Gy. However, at 40 Gy, the mean induced sterility (and upper and lower quartile) with 130 ml of water was higher than with 30 ml of water. The absence of significant effect of water quantity on pupal irradiation allows us to hypothesize that the amount of oxygen present in 30 ml of water is sufficient to irradiate 2000 pupae for a duration of 5,33 or 6,05 min to achieve 35 Gy and 40 Gy respectively. Further experiments with the measurement of oxygen depletion would allow us to test this hypothesis. However, in the context of a facility, and with the parameters of the irradiator tested on La Reunion, we recommend using the largest quantity of water, i.e. 130 ml, with the assumption that this provides a larger quantity of available oxygen to prevent total oxygen depletion, even if there is a delay in the irradiation procedure. Future experiments will test this hypothesis. Furthermore, the use of a large petri dish does not add complexity to the handling of the pupae during irradiation and doesn't increase handling time.

The optimal irradiation dose for *Aedes albopictus* pupae has been reported to be between 35 and 42 Gy in water^20,24,29,30^. It is important that insects used for the SIT are not over-dosed, as this can lead to a decrease in male biological quality^27^. We thus started with the lowest doses of 35, and 40 Gy to assess whether we could achieve the target sterility in the mosquito strain, and irradiation set-up used. We found that 40 Gy was more reliable in reaching the target sterility (above 99%). Previous studies have shown that survival of male pupae and flight ability in adults were not affected by increasing the dose up to 50 Gy^20,24^. In our experiment and with our irradiator, 40 Gy clearly appeared to be the best irradiation dose for upscaled irradiation of pupae, per batch of 2000 pupae in water.

Previous experiments have highlighted factors that affect dose response in pupae sterilised at small scale. In the context of optimizing the production of sterile males in a factory, we wanted to maximize pupae density, presenting a new factor. We found a difference between the irradiation of 2000 and 2500 pupae in 130 ml of water at 40 Gy. Thus, it was not possible to increase the density of irradiated pupae without effects on the irradiation outcome. A decrease in radiation effects as pupal densities increase may be correlated to the decrease in DO as more pupae respire in the available volume of water. With the Reunion irradiator specificities, we could thus irradiate only 14 000 pupae per irradiation cycle, a result that was used to plan and implement field releases of sterile males within our control program.

By testing the induced sterility of males irradiated at various locations in the canister/petri dishes, we found that overall, there was a good dose uniformity, and only position 7 had a slightly higher dose (as seen by higher sterility), which corresponds to the dose map performed during irradiator maintenance (Supplementary Fig. S2). Quality assurance is an important part of any successful SIT program for area-wide integrated pest management^19^.

Standardizing the sterilization process for mosquito pupae remains a challenge, and the irradiation at adult stage is becoming the preferred method. Irradiation protocols for adults are under development and require immobilization prior to compacting and packing the samples, either by chilling or the use of anaesthetics such as nitrogen^39,40^. However, the sterilization method defined by highlighted factors in previous experiments showed consistent and reproducible results that can be used to irradiate pupae, thus offering an alternative to countries considering pupae irradiation. With these settings, the target of 300,000 sterile males per week could be fulfilled with a total irradiation time of about 2h20 per week. If such an irradiator was fully available to the SIT program, the upscaled irradiation protocol presented here may allow producing more than 5 million sterile males per week considering 5 working days of 8 h.

## Methods

### Strain

The *Aedes albopictus* strain used for the experiment originated from field egg collections in the northern region of Reunion Island at Saint-Marie in 2014. The strain has been maintained under laboratory conditions at the CYROI, Saint-Denis, Reunion Island, in a climate-controlled insectary at 27 ± 2 °C, 75 ± 2% relative humidity (RH), photoperiod of 12: 12 h (light: dark).

### Adult rearing

Adults were kept in standard rearing cages (30 × 30 × 30 cm, Bugdorm, Taiwan) composed of 1125 females and 375 males with continuous access to 5% (wt:vol) sucrose solution. After having been deprived of sugar for 5 h, females were offered blood meals with defibrinated fresh bovine blood for 30 min using the Hemotek® membrane feeding system (plate of 10 cm diameter and 20 ml total blood capacity). Egg collections were performed two days after blood meals. Females oviposited in plastic beakers lined with crepe paper (Sartorius Stedim Biotech GmbH, Göttingen, Germany) containing deionized water and placed in the cage. Oviposition cups were removed and filter papers were left to dry for one week in plastic containers covered with an opaque plastic plate at the ambient conditions of the insectary.

### Hatching

Egg papers from one to three months old were placed in 250 ml plastic jars with 225 ml of water for 30 min. 25 ml of hatching solution (0,3% wt:vol acid ascorbic solution) was added to the jars which were then closed for 4 h to allow the optimal decrease of oxygen for hatching. A larvae counter was used to quantify hatched larvae into batches of 4000 larvae, which were then transferred to plastic trays (30 by 60 by 10 cm) containing 2 L of water. The trays were covered and larvae were fed daily for 4 days with a total of 100 ml of development solution^15^. Pupae were collected daily, sexed on pupal size dimorphism using a glass pupal sorter and transferred into plastic beakers inside a fresh adult cage for emergence.

### Sample preparation

All male pupae used in the following experiment were collected until 30 h before irradiation, which means pupae were 30–44 h old. Pupae were sexed based on pupal size dimorphism using different sizes of sieves in water and sex was verified under a stereomicroscope. Females were placed in individual tubes for emergence to ensure sex and virginity for later mating. Cups of male pupae were transported to the irradiation facility and placed in petri dishes just before irradiation. For tests with water, renewed and temperate tap water was used in petri dishes. Several repetitions were performed according to the experiments as described below.

### Irradiator

The X-ray irradiator (Actemium Cegelec, BloodXrad 13–69, Le-Plessis-Pâté, France) of the CHU Félix Guyon (Saint-Denis, La Réunion) was used for this experiment. It is the only irradiator on the island. It was programmed to deliver a dose of 35 Gy or 40 Gy for each exposure, with a dose rate of 0.11 Gy/second. A plastic cylindrical box (14 cm high with a diameter of 14.5 cm) served as the irradiation canister, corresponding to 2312 cm^2^. Petri dishes were stacked inside the canister to increase available surface area for pupae The irradiation chamber and canister are positioned between 2 X-ray tubes (above and below). The irradiator dose rate is verified every year at 15 positions in the canister using an ionization chamber (TM31010) coupled with a UNIDOS electrometer (UNIDOS PTW T10021 n° 000778, Freiburg, Germany) (Supplementary Fig. S2).

### Effect of exposure environment and pupae density

The effect of exposure environment and pupae density during irradiation has been assessed and reported for two exposure environments (water and air) and for five pupae densities (500, 2000, 3000 and 4000). Male pupae were placed in a petri dish (8.5 cm × 1.2 cm). For the water environment, 30 ml of tap water was added with 500, 2000, 3000 and 4000 pupae, corresponding to pupae densities of 16.7, 66.7, 100 and 133.3 pupae /ml respectively. For the air environment, a hole (5.8 ± 0.1 cm) was made in the center of the petri dish, covered with a net to allow water to escape. Male pupae were placed in the petri dishes and excess water was removed by placing the petri dishes on an absorbent paper (but not dried). Thus, the pupae were in the air, but still in damp conditions. For irradiation, 5 to 8 stacked petri dishes were placed in the center of the canister and pupae were irradiated with 35 Gy.

### Effect of dose and water quantity

The effect of dose and water quantity during the irradiation of 2000 pupae has been assessed and reported for two doses (35 Gy and 40 Gy) and for two quantities of water (30 ml and 130 ml). Classic petri dishes (8.5 cm × 1.2 cm) were used for 30 ml, corresponding to 66.7 pupae/ml; and large petri dishes were used for 130 ml (13.7 cm × 1.7 cm), corresponding to 15.4 pupae/ml. In both petri dish types, water surface was sufficient to allow all of the pupae to remain in a monolayer and to have access to air with sufficient space around them. For each petri dish, pupae remained in a single layer on the surface of the water. For irradiation, 5 to 8 classic petri dishes, or 4 to 7 large petri dishes were stacked and placed at the centre of the canister.

### Optimization of pupae density

According to the results of experiments 1 and 2, we determined the most suitable exposure environment, pupae density, dose and water quantity for upscaling the irradiation procedure of male pupae of *Ae. albopictus*. In the context of optimizing the production of sterile males in a factory, we wanted to test whether the use of these two factors (dose and water quantity) could optimize the number of pupae irradiated per petri dish, i.e., the irradiation of more than 2000 but not exceeding 3,000 pupae. The effect of pupae density during irradiation in water at 40 Gy and in large petri dishes (corresponding to 130 ml of water, or 15.4 pupae/ml) has been assessed and reported for 2 pupal densities (500 and 2000) and an intermediate pupal density (2500). For irradiation, 5 to 7 large petri dishes were stacked and placed at the center of the canister and pupae were irradiated with 40 Gy.

### Effect of the pupae location within the irradiation canister

The effect of the location of pupae during upscaled irradiation has been assessed and reported for 7 positions within the petri dishes, corresponding to the location of the petri dishes in the canister, from position 1 at the bottom of the canister to position 7 at the top of the canister. In this experiment, the canister was fully loaded. For irradiation, 7 large petri dishes were stacked and set in the canister and pupae were irradiated with 40 Gy in 130 ml of water.

### Assessment of induced sterility

Combined effects of all factors assessed in the previous 4 experiments were determined based on the induced sterility of adults. For each experiment and repetition, a control was provided with non-irradiated male pupae. For each test and control, 150 male pupae were randomly selected and transferred to plastic cups inside a fresh adult cage for emergence with a continuous access to 5% (wt:vol) sucrose solution. 150 virgin females were added to the cage. After 9 days, a fresh blood meal was offered to females over two consecutive days and oviposition cups were placed in the cages four days after blood feeding (see adult rearing section). Oviposition cups were removed after 32 h and the filter papers were left to dry for one week in plastic containers covered with an opaque plastic plate at ambient conditions of the insectary. Eggs were hatched after 17 days (see hatching section). The number of eggs that hatched out of the total number of eggs produced (fertility) was determined over a single gonotrophic cycle under a stereomicroscope. On each paper, 3 bands of a minimum of 350 eggs were counted, at the left, the center and the right. Preliminary analyses have shown non-significant differences between bands for all tests or controls (Generalized linear mixed model fit by maximum likelihood (Laplace Approximation), *P* > 0.7950). In the study, experimental methods changed between experiments as new information regarding certain parameters became available, leading to some variations in sample size and number of repetitions between the different experiments.

### Statistics

Induced sterility (IS) was calculated for each treatment group as follows: IS = (HR_c_ − HR_t_)/HR_c_; where HR_t_ is the hatch rate of the treatment (t) group, and HR_c_ is the hatch rate of the control (c) group. The overall natural (control) fertility of the strain used was 0.92 (± 0.03) in 96 reps, but controls between experiments showed differences, thus we chose to use ponderation to analyse data. Each test IS had been divided by its associated control IS. Statistical analyses were performed using Microsoft Excel (v.16.0, Microsoft, Redmont, WA, USA) and R v4.2.3^41^.

The effect of exposure environment and pupae density during upscaled irradiation on induced sterility was analyzed using a gaussian linear mixed-effects model fit by maximum likelihood, with exposure environment, pupae density and their first order interaction used as fixed effect and repetition as a random effect. The same analysis was used to test the effect of irradiation dose and water quantity during upscaled irradiation on induced sterility, but without a significant interaction between the two fixed effects.

The effect of the optimization of pupae density during irradiation on induced sterility was analysed using a gaussian linear mixed-effects model fit by maximum likelihood, with location as a fixed effect and repetition as a random effect. The same analysis was used to test the effect of location in the entire canister during irradiation on induced sterility.

The best model in all analyses was selected based on the lowest corrected Akaike information criterion (AICc), and the significance of fixed effects was tested using the likelihood ratio test^42,43^. All significant differences are based on *P* < 0.05.

### Ethical approval

No specific permit was required for the study reported herein. The cow blood used for mosquito feeding was handled in strict accordance with the health regulations and guidelines of the local Animal and Environmental Health Protection protocol (Ref. ANI.ENV.ENR 002) and in compliance with the authorization issued by a Prefectural decree No. SALIMPSPAE -2021-214-D for the use of animal by-products for research purposes.

### Supplementary Information

Supplementary Figures.

## Data Availability

The data generated and analyzed during the current study are available from the corresponding authors on reasonable request.
