# Supplementary material for: Upscaling irradiation protocols of Aedes albopictus pupae within an SIT program in Reunion Island
Source: Sci Rep. 2024 May 27;14:12117. doi: 10.1038/s41598-024-62642-7 (PMC11130285; doi:10.1038/s41598-024-62642-7)
Supplement: Supplementary file 1 — Supplementary Figures. [file 41598_2024_62642_MOESM1_ESM.docx]

# Supplementary Figures


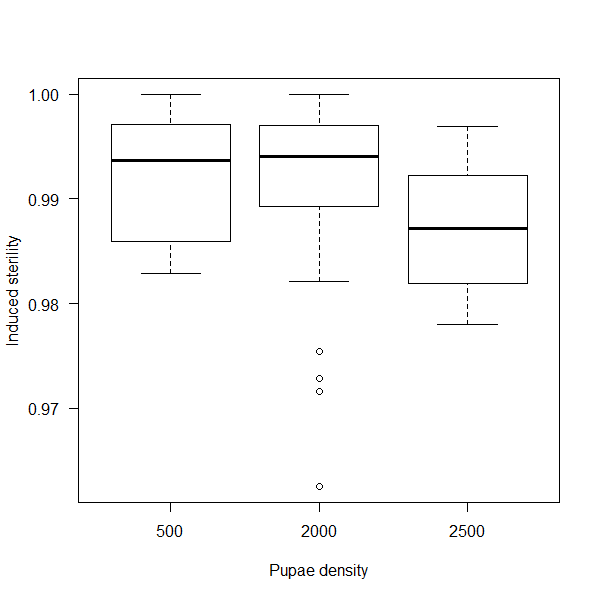


**Supplementary Figure S1.** Induced sterility of *Ae. albopictus* following irradiation of increasing pupae densities with 40 Gy and with 130 ml of water. The box plot shows the median and upper and lower quartiles.


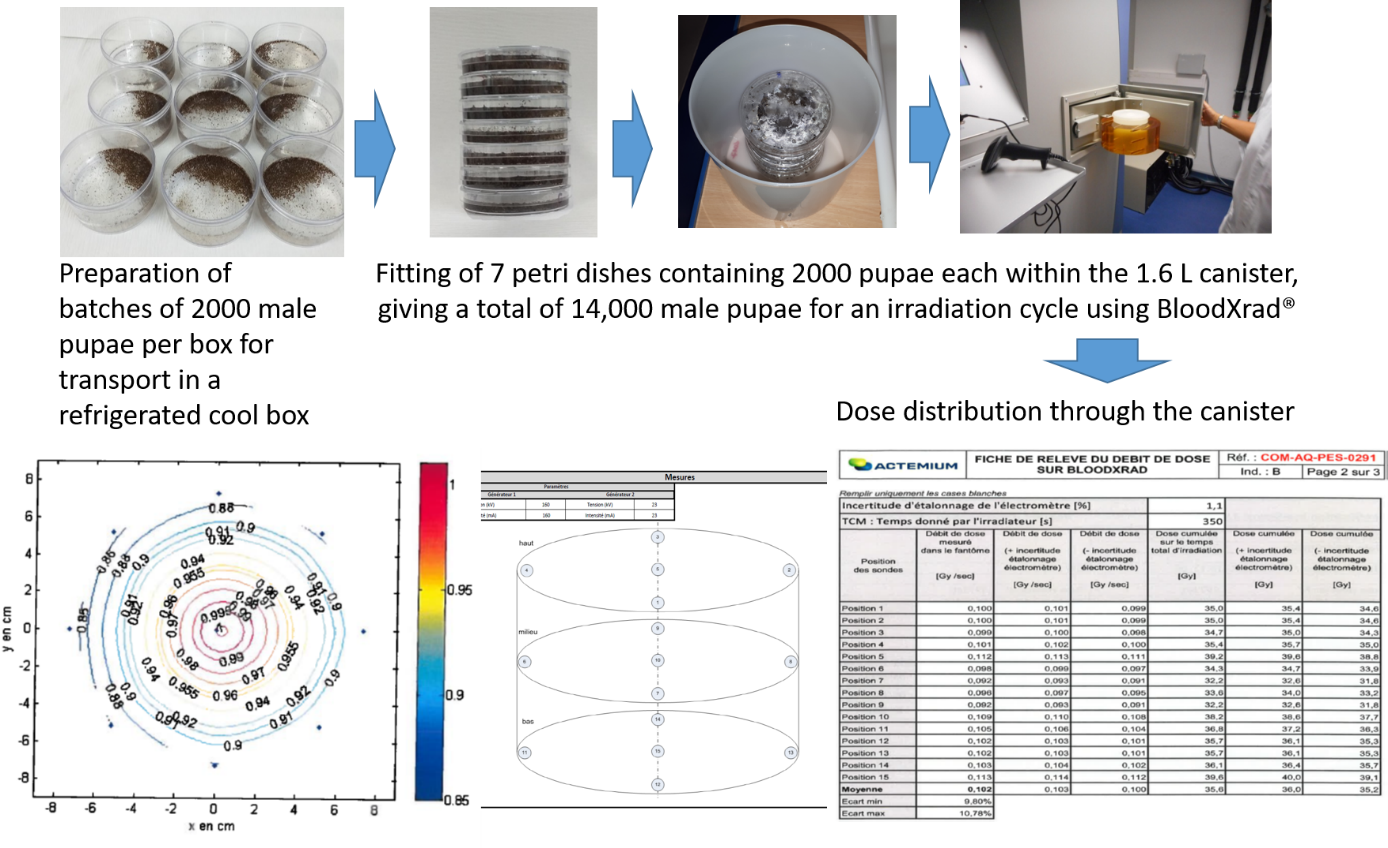


**Supplementary Figure S2.** Irradiation protocol – Canister setup. In practice, the mosquito sample is placed in the canister. Dose mapping within the full volume of the canister in the X-ray irradiators involved the use of alanine dosimeters. The average dose rate of the all reference points was 0.102 Gy/sec (range: 0.92 – 0.113). The DUR calculated was 1.7 for the whole canister.
